# Coping Strategies Used by Older Adults to Deal with Contact Isolation in the Hospital during the COVID-19 Pandemic

**DOI:** 10.3390/ijerph18147317

**Published:** 2021-07-08

**Authors:** Jogé Boumans, Aukelien Scheffelaar, Vera P. van Druten, Tessel H. G. Hendriksen, Lenny M. W. Nahar-van Venrooij, Andrea D. Rozema

**Affiliations:** 1 Tranzo, Tilburg School of Social and Behavioral Sciences, Tilburg University, 5037 AB Tilburg, The Netherlands; a.scheffelaar@tilburguniversity.edu (A.S.); v.v.druten@jbz.nl (V.P.v.D.); a.d.rozema@tilburguniversity.edu (A.D.R.); 2 Jeroen Bosch Academy Research, Jeroen Bosch Hospital, 5223 GZ ‘s-Hertogenbosch, The Netherlands; T.Hendriksen@jbz.nl (T.H.G.H.); l.nahar@jbz.nl (L.M.W.N.-v.V.)

**Keywords:** COVID-19, older adults, contact isolation, coping strategy, realist evaluation, hospital setting

## Abstract

Due to the COVID-19 pandemic, many older adults have experienced contact isolation in a hospital setting which leads to separation from relatives, loss of freedom, and uncertainty regarding disease status. The objective of this study was to explore how older adults (55+) cope with contact isolation in a hospital setting during the COVID-19 pandemic in order to improve their physical and psychological wellbeing. The realist evaluation approach was used to formulate initial program theories on coping strategies used by (older) adults in an isolation setting. Twenty-one semi-structured interviews with older patients (*n* = 21) were analysed. This study revealed that both emotion-focused coping strategies as well as problem-focused coping strategies were used by older adults during contact isolation. The study also uncovered some new specific coping strategies. The results have useful implications for hospital staff seeking to improve the wellbeing of older adults in contact isolation in hospitals. Problem-focused coping strategies could be stimulated through staff performing care in a person-centred way. Trust in staff, as part of emotion-focused coping strategies, could be stimulated by improving the relationship between patients and staff.

## 1. Introduction

Contact isolation is often an unpleasant experience. Isolation is characterised by the separation from loved ones, the loss of freedom, uncertainty over disease status, and boredom [1,2,3,4]. Patients in contact isolation are more likely to develop symptoms of depression and have longer lengths of stay than non-isolated patients [5,6]. Additionally, when compared with non-isolated patients, physicians and nurses have been shown to have fewer direct interactions and perform fewer examinations on isolated patients [7,8,9]. Patients in contact isolation have reported a poor understanding of the reasons and procedures for contact isolation and a greater level of dissatisfaction with their care as a result [10,11].

Coping strategies, in the form of behavioural and cognitive efforts, are used by individuals to deal with a variety of stressful situations in order to lower the levels of stress [12,13,14]. For this study, the theory of Richard Lazarus and Susan Folkman regarding different coping strategies was followed because much of the literature about coping behaviour of older adults [15,16,17,18] is based on their theory that two different coping strategies can be distinguished, namely problem-focused and emotion-focused coping [19,20,21]. Other works such as Carver and Scheier’s multidimensional coping strategies have been used mainly for other target groups and were therefore not selected for the current study.

Problem-focused coping includes those strategies that involve different mechanisms like acting on the environment (e.g., seeking support from others to solve the problem) or the self (e.g., cognitive restructuring) to modify the problem at hand [21]. Problem-focused coping strategies typically include elements such as generating options to solve the problem, evaluating the pros and cons of different options, and implementing steps to solve the problem [21]. There are several studies on the use of problem-focused coping strategies in relation to contact isolation in hospital settings [22,23,24]. Various studies showed that contact isolation affected patients’ feelings regarding the sense of control over their health [25,26,27], isolation situation [4,23,27], and their day- to-day living in isolation [23,26,28,29]. Providing patients with more control over their health (for example by providing sufficient information [30]), isolation situation (for example by including patients in decisions [27]), and/or day-to-day living situation (for example by providing a radio or a clock [25]) could trigger problem-focused coping strategies. 

Emotion-focused coping does not address the problem at hand; however, it deals with the feelings and perceptions associated with the stressful situation and includes those strategies used to regulate one’s stressful emotions [21]. The range of emotion-focused strategies is quite broad, including denial, the focusing on and the venting of emotions, the positive reinterpretation of events, and seeking out social support [31]. To our knowledge, there are no studies on the use of emotion-focused coping strategies with contact isolation in a hospital setting.

Studies show that the use of coping strategies changes over the course of life [32,33,34,35,36]. Folkman and Lazarus’s [21] transactional model of stress and coping propose that age differences in coping strategies could be the result of changes in what people must cope with as they age. According to other studies on the relationship between age and coping, younger adults use more problem-focused coping strategies and older adults use more emotion-focused coping strategies [16,34], such as distancing oneself from a negative event [18]. This could be related to the fact that increasing age is usually associated with increased chances of chronic diseases such as cancer, cardiovascular disease, Alzheimer’s disease, Parkinson’s disease, arthritis, diabetes, and obesity [37]. In addition, older adults may have the advantage of years of experience and wisdom [38].

Due to the COVID-19 pandemic, many older adults have had to experience a contact isolation situation in a hospital setting. To our knowledge, no study to date has been performed that considers how and why the care situation for older adults during a pandemic in a contact isolation hospital setting triggers coping strategies. We performed a study on this specific care situation for older adults using the realist evaluation approach in order to improve care processes and the care environment. Realist evaluation was chosen as a research approach as it is designed to answer the why and how questions regarding the coping behavior of older adults while also taking into account the specific care context of the contact isolation situation in a hospital setting.

## 2. Materials and Methods

A qualitative study was performed to explore the experiences of older adults in a contact isolation setting and to identify the coping mechanisms applied. First, the theoretical framework based on realist evaluation is described, followed by the program theories. Thereafter, the selection of the participants, the interview, and the analysis are described.

### 2.1. Theoretical Framework

This study was informed by the realist evaluation approach [39]. Realist evaluation is a theory-driven method and examines what works for whom, in what context, to what extent, how, and why [39]. A realist evaluation describes not only the intervention and its outcome (O), but also the context (C) and the underlying mechanism (M). This is performed by discerning the psychosocial mechanisms (not directly observable and which include preferences, reasoning, norms, or the collective belief of people) that trigger intervention outcomes (changes to people and to their lives, which also includes other kinds of alterations) in specific contexts (including elements such as the organisational context, participant features, staffing, and the geographical and historical context of implementation) [40]. In the current study, the intervention was the contact isolation setting, the context was the hospital setting during a pandemic, the mechanisms were the coping strategies used by older adults, and the outcome was the influence of the use of coping strategies on the physical and psychological wellbeing of older adults.

A realist evaluation begins with the formulation of the theory behind the development of an intervention, known as the initial program theory. The function of the initial program theory is to describe and explain, as much as possible, how and why the program (i.e., the intervention) may be working for some people and not others, depending on which mechanisms are or are not triggered in specific contexts. The program theory is formulated on the basis of previous research and/or knowledge and the experience of stakeholders involved in the intervention. At the start of this study, scientific literature about contact isolation (in a hospital setting), coping strategies, and coping strategies used by (older) adults were searched, synthesised, and discussed between the first author (J.B.) and the last author (A.D.R.). Based on this process, the initial program theories were established (see Table 1). Realist evaluation involves theory testing and refinement; therefore, the results found during this study were used to refine the initial program theory [41]. RAMESES II reporting standards for realist evaluations were followed [42].

### 2.2. Participants

Respondents were approached by a doctor in the outpatients’ clinic for post COVID-19 patients approximately 6 to 8 weeks after being discharged from the hospital. In total, 221 patients were eligible for participation; 156 patients agreed to be contacted by the researchers were invited for an interview and received written information. Of these, 138 patients provided their informed consent, and 32 patients agreed to give an interview. Of those 32 participants, 21 participants met all the inclusion criteria for the current study. The following inclusion criteria were used to select participants for this part of the study: (1) 55 years or older; (2) capable of verbally communicating in Dutch; and (3) a minimum stay of two days on the COVID-19 ward. Patients were excluded from the study if they were admitted to the intensive care ward (receiving more than 25 L of oxygen per day) or if they were diagnosed with dementia. Patients who experienced a delirium during hospitalisation were included unless the researchers concluded that their memories were too limited, making them unable to answer the questions.

All the interviews were conducted by one researcher (V.P.v.D.) who was coached and supervised by the two last authors (L.M.W.N.-v.V., and A.D.R.). Interviews were conducted by telephone and took place between June 2020 and September 2020. Informed consent was initially given verbally (by phone) and confirmed at the time of the interview. Sociodemographic data were obtained from the participants’ electronic patient files.

### 2.3. Data Collection Setting

Semi-structured interviews with older adults focusing on the patients’ experiences of COVID-19 care were conducted in a multidisciplinary teaching hospital in the southern part of the Netherlands. At the start of the COVID-19 pandemic, the outbreak was the most severe in this part of the Netherlands [45]. This hospital is one of the largest in the region with 630 beds [46]. The current study concerned a secondary data analysis of data collected for a larger study, ‘Consequences of COVID-19 care’.

### 2.4. Interview

Semi-structured interviews were performed using an interview guide including optional probing questions for clarification to explore the experiences of the respondents receiving COVID-19 care in contact isolation in the hospital. The interviews lasted between 35 and 90 min. The interview guide was based on the four constructs of the framework for person-centred nursing, which describes prerequisites for person-centred practice [47] and on the taxonomy of patient preferences for patient-centred healthcare [48]. The topics of the framework for person-centred nursing are: (1) the attributes of the nurse (professional competence, interpersonal skills, commitment to the job, and personal characteristics); (2) the care environment (the context in which care is delivered); (3) person-centred processes (caring as perceived by patients and including providing for patients’ physical and psychological wellbeing needs); (4) the results of effective person-centred nursing (feeling of wellbeing (affective and physical), patient satisfaction, and the effect on the environment) [47]. The topics of the taxonomy of patient preferences are: (1) Patient Uniqueness (individual human beings with their own life stories, history, culture, and backgrounds); (2) Patient Autonomy (personal decision to participate in decision-making or not); (3) Professional Compassion (personal touch/disclosure); (4) Professional Professionalism (open to reflection and professional collaboration); (5) Professional Responsiveness (committed and responsible execution of care); (6) Interaction Partnership (involvement in planning and (shared) decision making); and (7) Interaction Empowerment (ability to contribute to self-management and trust) [48]. The questions were designed to uncover the open feelings and thoughts of respondents regarding their experience with COVID-19 care in the contact isolation situation, without a direct relationship with the initial formulated program theories. All interviews were recorded and transcribed verbatim. Table A1 provides the translated semi structured interview guide.

### 2.5. Analysis

Only the parts of the interview that referred to the contact isolation situation were analysed. Interviews were audiotaped, transcribed, and analysed applying the Context–Mechanism-Outcome configurations (CMO configurations) as the analytical tool [49,50]. Identifying essential similarities and differences between experiences of older patients enabled the grouping and aligning of CMO configurations to identify patterns guided by the following questions: what had an impact on patients’ physical and psychological wellbeing (outcome)? What (if possible, which coping strategies) caused these effects (mechanisms)? What where the isolation circumstances when these effects occurred (context)? The goal of the analysis process was to identify CMO configurations that underpin the initial program theories about coping strategies used by (older) adults in an isolation setting and to identify CMO configurations that may lead to new insights about coping strategies and changes in the initial program theory.

The transcripts were imported in the qualitative data analysis software program Atlas-Ti for data analysis [51,52,53]. Based on the initial program theories, a code tree was drafted by JB on possible coping strategies. Coding was based on the link between context-mechanism-outcome and the performed or intended coping strategies. Researchers (J.B. and A.S.) independently coded two interviews and discussed differences in interpretations to establish and refine the code tree. Thereafter, the first author coded the remaining interviews independently and discussed the sections which she was not certain how to code with the second author. To increase the inter-researcher reliability, a check was performed by the second author. She independently coded two and randomly selected other transcripts during the coding process of the first author and the coded sections were compared. After discussion, extra codes that had emerged from the data were added. The main findings were discussed by the first two authors and a structure was determined to report on the main findings.

### 2.6. Ethics

The study was approved by the Medical Research Ethics Committee Brabant (MREC Brabant) (NW2020-42). Participants were given both written and verbal information about the study, including the purpose, confidentiality of interviews, the voluntary nature of participation, and the opportunity to withdraw at any time. Interested participants took part after providing verbal consent at the beginning of the interview.

## 3. Results

The results section starts with the description of information on the setting and respondents, presented in Table 2 This table provides an overview of the two settings which were studied, the Suspected COVID-19 Ward and the Cohort COVID-19 Ward. In the results, the specific setting was identified but no comparison between the two settings was made. Twenty-one older adults participated in the present study; their background characteristics are provided in Table 3. Thereafter, the findings are described in detail following the structure of the initial program theories. 

### 3.1. Problem-Focused Coping Strategies

#### 3.1.1. Approach of Staff

One of our program theories focuses on the approach of staff. We studied two existing mechanisms on the approach of staff which were already distinguished in the literature: (A) staff personalised approach and (B) provision of information to the patient. Additionally, two new mechanisms came to the fore: (C) professional competence of staff and (D) the provision of information by staff to family members.

(A) Staff personalised approach

We hypothesised that a personalised approach of staff could be an important mechanism for coping among older patients. We found four explanations for this theory. First, patients on the suspected and on the cohort ward explained that they noticed that staff spent time with—and paid attention—to them on a personal level. For example, staff did not only ask about the health of the patients but also how the patients were dealing with the whole situation of being in the hospital. This made the patients feel that they were being treated as people rather than numbers. Second, on some occasions, both on the suspected and on the cohort wards, staff would sit at the patients’ bedsides to talk with them about their feelings or reassure them when they were feeling emotional. For example, on one occasion on the suspected ward, a staff member just sat next to the patient who expressed feelings of panic. Just by being present, the staff member reassured the patient and the patient’s breathing returned to normal.

*‘I was so short of breath and of course I was lying alone and then she sat with me for an hour, really for an hour. Then she sat with me, just sat, I was lying down [on the bed] of course………I was short of breath, she said nothing else, just sat, just being there, that was already quite something. I liked that.’*

(Respondent 5 suspected ward)

Third, staff took their own initiatives for (care) tasks and sometimes performed extra tasks besides the usual work, which patients did not expect. Several patients on the cohort ward explained that staff provided them with food and drink without them asking for it. Additionally, one patient provided the example of a care professional who printed out photos of the patient’s relatives and put them up in the patient’s room. This made the patient feel seen as a person. The fourth example of personalised approach was staff starting reciprocal conversations with patients about topics not related to caregiving, such as conversations about their children or hometown.

Explanation of the coping: staff spent time with—and paid attention to—patients on a personal level, they took time to sit at the bedside to talk with the patients, they took their own initiatives with regard to (care) tasks, and had reciprocal conversations with patients about topics not directly related to caregiving (mechanism). This personalised approach made individual patients feel treated as a person (coping).

(B) Provision of information to the patient

One of the hypotheses based on the coping literature concerned the provision of information by staff. This could be an important mechanism for coping strategies of older patients because it provides patients with certainty and/or an understanding of the situation. The findings confirmed the relevance of information provision for the coping mechanisms of patients in the cohort ward. Open and honest communication by the staff about the medication and about the health circumstances (e.g., level of oxygen, relapse in health condition) of the patients gave patients a feeling of trust. Although this open style of communication was not always felt to be pleasant or easy to hear by the patients, it helped them to understand the situation.

*‘Interviewer: And the doctor had also said to you: we can’t do anything for you. Respondent: Yes. Interviewer: How did you feel about the doctor saying that? Respondent: Well, (…) that came across in a harsh way. (…) Interviewer: And how did you experience that? Respondent: Yes, he said it, and he explained it. So that was a shock (…) but it was also immediately explained, and I understood it. So, it was a shock for a moment and then it was over.’*

(Respondent 1 cohort ward)

Explanation of the coping: open and honest communication by the staff about the (health) circumstances of the patients (mechanism) gave the patients a feeling of trust and helped them to understand the situation (coping).

(C) Professional competences of staff

A new mechanism regarding the approach of staff was found during the data analysis which was not yet identified in the literature. Patients in the suspected ward and in the cohort ward explained that they relied on the professional competences of the staff, related to two specific approaches. First, several patients reported that the staff were conducting their work in a very professional way (e.g., in a very routinised and competent, self-aware way of operating, showing significant commitment) and second that staff members were keeping a close eye on the patient by taking regular measurements (e.g., asking patients how they felt and measuring blood pressure and oxygen level). This made the patients feel closely monitored by professionals and, therefore, safe.

*‘And they performed all the various measurements, so sometimes about five times a day they came by to measure this, measure that, and that gave a certain, yeah a safe feeling, you thought you were well monitored.’*

(Respondent 21 cohort ward)

Explanation of the coping: professional competences of staff and regularly monitoring checks upon the patients (mechanism) made the patients feel looked after and safe (coping).

(D) Provision of information from staff to relatives

Another new mechanism regarding the approach of staff was found during the data analysis: the regular contact with—and information provision to—relatives. Patients on the cohort ward mentioned that the staff had daily contact with their relatives about their health situation and to arrange the delivery of personal items. It was comforting for the patients to know that their relatives were informed regularly, especially because some patients were not able to contact their relatives themselves due to the condition of their illnesses.

Explanation of the coping: awareness that of the fact that relatives were kept up to date about the health situation of the patient by the care professional who also made practical arrangements (mechanism) made the patients feel reassured and comfortable (coping).

#### 3.1.2. Sense of Control

One of our theories focuses on the control that patients have over their health, isolation situation, and day-to-day living situation. We looked to two existing and two new mechanisms of this approach for the use of problem-focused coping strategies of older patients: (A) patient involvement in decision-making by staff; (B) patients taking the initiative on shared decision making (a new mechanism); (C) the provision of personal items or a private room; and (D) the increased recognisability of staff (a new mechanism).

(A) Patient involvement in shared decision making

Both patients from the suspect ward and from the cohort ward described situations in which they were included in decisions regarding day-to-day living situations. In one instance, a patient on the suspect ward wanted his bed to be placed in a different direction. In another instance, a patient on the suspect ward wanted to sit on a chair. In both instances, the staff responded to their needs; the bed was replaced, and a chair was brought to the room. Patients of the cohort ward also gave examples of staff actively asking them about the light settings or about placing photos of their relatives in the room. This would make the patients feel more at ease.

On the cohort ward, staff included patients in decision making for both medical or disease-related decisions (e.g., health status changes, increase or decrease in oxygen supply) and preferences regarding quality of life (e.g., diet choices, timetable, and activities related to physical care). An example showed the importance of communicating clinical guidelines to patients. In one instance, the patient wished for an extra opiate to relieve the pain which was not given by the staff with the explanation that a certain schedule was retained. The explanation helped the patient understand why his wish was not granted. On the suspected ward, very few examples of including patients in decision making regarding their health and isolation situation were found.

Explanation of the coping: when staff included patients in decision making regarding their health or regarding day-to-day living situations through explanation or by actively asking patients about their preferences (mechanism), this could make the patients feel in control of health or day-to-day living situation (coping). Conversely, some patients trusted staff and relied on them to make decisions about their health without expressing the need to be in control of their own health situations.

(B) Patients taking the initiative on shared decision making

A new mechanism regarding sense of control was found during the data analysis: patients on the cohort ward occasionally took their own initiative to be included in decisions regarding their own health. Patients asked staff for information regarding their own health (e.g., to provide them with information regarding health procedures or if they could be discharged earlier) and asked staff to perform tasks like measuring their oxygen saturation or providing extra nutria drinks. This made the patients feel as they were taken seriously by the staff and made them feel listened to.

*‘I say: please take the ears. Then they [the staff members] took the fingers and the ears (…) [For measuring the oxygen level in the blood] So of course I had a say in that. Interviewer: And how was this responded to by the care professionals Respondent: Right away. They also did it right away and they also looked at it and yes, it was just well responded to. Interviewer: And what kind of feeling or idea did that give you? R: Well, also a safe feeling. They listen to me.’*

(Respondent 10 cohort ward)

Explanation of the coping: when patients indicated a desire to be included in decision making regarding medical decisions by requesting staff for information or requesting specific care tasks and when staff respond to these requests (mechanism), this made the patients feel in control of their health situations.

(C1) Provision of personal items

During their stay at the suspect ward and the cohort ward, the patients described situations in which they felt more at ease because they had access to personal items, like a tablet, laptop, or a television. This made their stay less monotonous, because they were in control over what they wanted to see and hear. For the patients in a single room, personal items like the television and the tablet gave them the opportunity to turn on music when they felt the need to do so. Patients sometimes used the video conference option on their devices; this is described in more detail in the emotion-focused coping strategy ‘seeking support from family and friends’.

Explanation of the coping: when patients have access to personal items (mechanism) they could have more opportunities to control their day-to-day living situation in an isolation situation because they are in charge of what they wanted to see or hear (television, tablet, laptop).

(C2) Provision of a single room

The preconceived situation in the hospital where this study took place was that patients on the suspect ward would be placed in a single room and that patients on the cohort ward would be placed in a room with more persons. Several patients on the suspect ward were content with the single room because they were very ill and appreciated the calm environment. Some patients thought it was a normal procedure in case of an infectious disease and had no trouble with this situation. One patient expressed feelings of loneliness.

The patients of the cohort ward described positive and negative responses towards a shared room. A positive effect was the support and company of other patients. A negative effect was not having a calm environment and also sometimes experiencing frightening situations with other patients (e.g., patients who were suffering from breathing troubles or patients who were passing away). The theory that a single room could help older patients cope with a contact isolation situation could not be confirmed by our findings nor could it be refuted.

Explanation of the coping: the benefits of having a single room could be related to the preferences of the patient. If a patient preferred a single room (mechanism), this created a calm environment for the patient in which they could focus on their own health (coping). However, when a patient did not prefer a single room, this led to loneliness.

(D) Increased recognisability of staff

A new mechanism regarding sense of control came to the fore. Patients from the suspect ward and from the cohort ward explained that the protective clothing (apron, gloves, mouthguards, hairnet, goggles) of the staff made patients feel uncomfortable. The staff were difficult to recognise which made it difficult to connect with the staff and caused the care to be perceived as anonymous. The patients on the cohort ward observed that some staff wore name tags or a photo of themselves on their clothing to increase recognisability. This made it easier for patients to see the person behind the clothing and connect with staff.

Explanation of the coping: if staff dressed in protective clothing could be less anonymous by wearing name tags or photos of themselves (mechanism), this could help patients to recognise and connect with the staff members who were helping them (coping).

### 3.2. Emotion-Focused Coping Strategies

#### 3.2.1. Trust in Staff

One of our program theories was that if older adults felt confidence in the staff, this would help them cope better with the isolation situation. We found four explanations for this theory. First, patients did indeed state that they felt very confident because the hospital was the best place for them to be cared for. Second, this feeling was enhanced because patients felt closely monitored and regularly checked on each day. Third, feelings of trust were increased when staff paid attention to the circumstances of patients and when staff discussed and agreed on the treatment with the patient. Fourth, trust seemed related to the availability of staff, and patients indicated that staff members were easily reached when they called or asked for help.

*‘I just felt confident in the fact that they were keeping everything under control [the staff members] and were monitoring me very closely in all kinds of ways. That gives you confidence, that you are being treated well and that you are in good hands.’*

(Respondent 8 cohort ward)

Some of the patients, a number of whom felt very ill, took a more indifferent attitude: they let staff decide for them because they were confident that the best choices would then be made.

*‘I was like yes it has to happen anyway so yes they will know. So, that’s just because I didn’t know myself so, and yes I don’t know, yes I just let it happen, they know what they are doing’*

(Respondent 9 cohort ward)

Explanation of the coping: professional care provision, regular monitoring, attention of staff, and the availability of staff (mechanism) contributed to feelings of trust in the patients (coping).

#### 3.2.2. The Positive Evaluation of (Conflict) Situations

One of our program theories was that if older adults could positively evaluate conflict situations, this would help them cope better with their isolation situation. We did not find support or explanations for this theory in our findings.

#### 3.2.3. Seeking Support from Family and Friends

One of our program theories focuses on the support for patients by their family and friends. One existing and one new mechanism came to the fore: (A) seeking support from family/friends and (B) seeking support from fellow patients (a new mechanism).

(A) Seeking support from family/friends

Patients from the cohort wards described that contact with family and friends for support via their own mobile telephone and the opportunity to consult with family and friends gave them strength and reassured them. Patients sometimes used the video conference option. This made them feel less isolated and lonely. However, some patients also explained that they were feeling ill and that having contact with friends or family was too exhausting.

Explanation of the coping: being able to contact family and friends via a mobile device for support and to consult with family and friends (mechanism) gave patients strength, reassured them, and helped the patients to feel less isolated and lonely (coping).

(B) Seeking support from fellow patients

Some patients from the cohort ward were placed in a room with more people, leading to the emergence of a new mechanism. Patients reported that contact with fellow patients gave them the opportunity to talk about the situation and to realise that they were not the only one in the contact isolation situation.

*‘Interviewer: And what kind of feeling did that give you? R: Yes, some peace, that you are not the only one, that you are not alone.’*

(Respondent 5 cohort ward)

Explanation of the coping: contact with fellow patients, which provides an opportunity to talk about the situation (mechanism), helped patients realise that they were not the only one in the contact isolation situation (coping).

#### 3.2.4. Increased Impulse Control

One of our program theories focuses on older adults having increased impulse control. We did not find support or explanations for this theory in our findings.

#### 3.2.5. Acceptance and Rationalisation

A new program theory came to the fore. Acceptance and rationalisation were used as coping strategies by older adults in a contact isolation setting. Patients were able to put their situations into perspective. Some patients showed signs of resignation.

Many patients from both the suspected ward and the cohort ward explained ways in which they used acceptance and rationalisation as a way of coping with the situation. For example, some patients indicated that they understood that the staff had to wear protective clothing and therefore they accepted the consequences of these dress codes, namely the care professional being less recognisable. A second example was that patients indicated that they were affected, but they went beyond that negative feeling. For example, people indicated that they were aware of the fact that they were not allowed to leave the ward, but that they had to accept that situation.

*‘You’re located in a ward with large doors at the front of it, and red barrier tape and I didn’t feel confined though, but I mean you’re of cours, isolated, from the rest of the world. Interviewer: And what kind of feeling did that give you? Respondent: Yes, I am a man of freedom, so that is not pleasant, but on the other hand, there’s just no other way, it has to be this way, so that is resignation of some sort.’*

(Respondent 3 cohort ward)

A reaction which was also frequently identified was people reacting indifferently to the contact isolation situation. They indicated that they were too ill to be concerned about the consequences of contact isolation.

Explanation of the coping: by using acceptance and rationalisation (mechanism), patients understood the situation or accepted the negative feeling (coping).

#### 3.2.6. Downwards Comparison

Another new program theory that was found during the analysis concerned downwards comparison. A few cohort patients explained they were stimulated to think more positively about their own situations because they were confronted with other patients who were in worse conditions compared with their own situations:

*‘I thought, when I see them [other patients] lying there and they are getting even sicker. I thought to myself: boy, you shouldn’t be grieving at all because there are patients who are in a much worse condition then you are, so move on!’*

(Respondent 11 cohort ward)

Explanation of the coping: downwards comparison (comparing themselves with patients who were in worse condition) (mechanism) stimulated patients to think more positively about their own situations (coping).

Table 4 provides an overview of the program theories about coping strategies in this specific context and setting.

## 4. Discussion

This study revealed that both emotion-focused coping strategies as well as problem-focused coping strategies were used by older adults during contact isolation. Moreover, this study also provided insight into the specific ways in which older adults used problem-focused and emotion-focused coping strategies in contact isolation situations in a hospital setting.

This study showed that the coping strategies of the respondents were related to the behaviour of different groups of people. Some of the emotion- and problem-focused strategies were related to the behaviour of the patients themselves (problem-focused: patients taking the initiative for involvement in shared decision-making and emotion-focused: acceptance and rationalisation and downwards comparison). Other strategies were specifically related to staff behaviour (problem-focused: approach of staff and shared decision making and the emotion-focused: trust in staff). Emotion-focused coping strategies were also related to the behaviour of other groups, specifically relatives and fellow patients (support of family and relatives and support from fellow patients).

Coping strategies related to staff behaviour are important in order to improve the physical and psychological wellbeing of older adults in contact isolation in a hospital setting. The basis of these types of coping strategies is the reciprocal relationship between patients and staff members. Staff members are able to influence the use of these coping strategies. This influence is made visible when the program theories related to staff behaviour are compared with the framework of person centred-nursing [47] and of the taxonomy of patient preferences [48]. The program theory: the approach of staff is reflected in the construct of nurse attributes from the framework and in the constructs of the taxonomy, specifically Professional Responsiveness, Professional Professionalism, and Professional Compassion. All these constructs emphasise the importance of interpersonal skills and professional skills of the staff members providing person-centred care. Shared decision making is reflected in the construct of person-centred processes from the framework and in the constructs Patient Autonomy and Interaction Partnership from the framework. These constructs include communicating with the patient and sharing decision-making as an important element for providing person-centred care. The program theory trust in staff is reflected in the construct of person-centred processes from the framework and in the constructs Interaction Partnership and Interaction Empowerment from the framework. These constructs include elements that could improve the relationship between patients and staff members because staff members are providing for patients’ physical and psychological wellbeing needs. In other words, activities of staff members that qualify as person-centred care could also stimulate the problem-focused coping strategies of older adults when they are in a contact isolation situation in the hospital. Person-centred care for (older) patients is increasing in popularity and importance [54]. The current study contributed to this development by demonstrating that the coping strategies of older adults in a contact isolation situation in a hospital are also stimulated by person-centred care.

The findings showed that trust in staff is an important emotion-focused coping strategy. However, staff should be aware of the imbalances of power and invite patients openly for shared decision-making regarding healthcare decisions. Several studies address the imbalances of power in nurse-patient relationships that could increase the vulnerability and dependency of the patient [55,56,57]. Other studies showed that trust is an essential component of the relationship between staff and patients [30,43,58]. Moreover, the literature about the patient-staff relationship usually explains the fact that if patients trust staff, they are also better able to express themselves [59,60]. However, the current study results showed the opposite: because patients trusted staff members, they did not feel the need to be included in decisions. This extra dimension might be a relevant finding to consider in possible imbalances of power.

The strategy of acceptance and rationalisation was often mentioned in the findings. In a different study regarding acceptance as a coping strategy [61]. Two forms of acceptance as general coping reactions were studied. First, active acceptance (acknowledging a negative, difficult situation and dealing with it in a constructive way) had a positive effect. Second, resigning acceptance (not only to stop controlling the actual situation, but becoming passive in other areas of life as well) had a negative effect. The findings of the current study also suggested that older adults who showed active acceptance (acknowledging the contact isolation situation as a difficult situation but dealing with it in a constructive way) could cope with the isolation situation. This might support the findings of previous studies showing that older adults are better capable in their ability to control emotions [18,62]. 

In addition to the emotion-focused coping strategy of seeking support from family/friends, the strategy to seek support from fellow patients is a new one derived from our study’s findings. Traditionally, in contact isolation settings, patients are usually placed in a single room [3]. This could cause feelings of loneliness [3,63]. The current study revealed that an isolation situation in which patients are placed together might stimulate the use of an emotion-focused coping strategy, as the contact among patients might provide some distraction and opportunities for conversation. However, a shared room could lead to other problems like patients not having a calm environment and frightening situations like critically ill patients passing away. Therefore, it could be important to select those patients who are not too ill to be placed in a shared room (e.g., if the state of their illness is not too stressful for other patients).

### 4.1. Strengths and Limitations

This study had some limitations. In realist evaluation literature, an intermediate step is ideally included in which the initial program theories are discussed with stakeholders to verify them. Due to practical reasons, this check was not performed in the current study. Alternatively, the initial program theories are based on a thorough and extensive literature search on the use of coping strategies in (isolation) situations which supports the initial program theories in this way. Another limitation is that the study was conducted in one hospital. The inclusion of multiple hospitals could provide a more generic view on the care provided during the COVID-19 pandemic and the coping strategies used by older adults to cope with the contact isolation situation. A last limitation is that the study was based on patient experiences collected in retrospect on situations which took place three to five months prior to the interviews. The results could have been different if the older adults were interviewed during admission. This study provided unique insights and evidence as it reported on one of the toughest periods of the COVID-19 pandemic: just after the initial outbreak, when hospital care was under enormous pressure. Another strength is the thorough realist evaluation method used, as it is one of the first qualitative studies that provides explanations for why certain coping strategies work in contact isolation in a hospital setting.

### 4.2. Practical Implications

To improve the physical and psychological wellbeing of older adults in contact isolation in a hospital setting, it is important that emotion-focused coping strategies and problem-focused coping strategies are stimulated. Problem-focused coping strategies can be stimulated through staff performing care in a person-centred way, including taking the time to listen to patients, providing sufficient information, exercising shared decision making, and by increasing the recognisability of staff by adjusting clothing or by using a photo or a name tag. Emotion-focused coping strategies are more difficult to stimulate because they relate to intrinsic strategies to regulate one’s emotions of stress. Trust in staff can be stimulated by improving the relationship between patients and staff. However, future studies and implementation of the strategies may reveal whether these strategies are applicable and workable in other hospitals and target populations under isolation circumstances.

## 5. Conclusions

This study reported on coping strategies studied in a unique contact isolation situation in a hospital setting caused by the COVID-19 pandemic, which was explored just after the direct outbreak. Explanations of concrete applications of coping strategies were found through the use of realist evaluation. This study adds to previous literature which found that emotion-focused coping strategies are also used by older adults in this specific situation and provides new insights in the use of problem-focused coping strategies in this specific setting. In particular, problem-focused coping strategies can be stimulated through staff performing care in a person-centred way. Emotion-focused coping strategies are more difficult to stimulate because they are intrinsic strategies used by patients to regulate stressful emotions. Nevertheless, trust in staff is an emotion-focused coping strategy that could be stimulated by improving the relationship between patients and staff. These findings can be implemented by hospital staff to improve the wellbeing of older adults in contact isolation in hospitals in the future.

## Figures and Tables

**Table 1:** Initial program theories.

Context	Mechanism	Evidence	Outcome
**Problem-focused coping strategies**			
If older adults experience contact isolation in a hospital setting during a pandemic problem-focused coping strategies could be triggered by:	approach of staff: via the mechanisms: personalised approach Provision of information to the patient	[23,26,30]	This will impact their physical and psychological wellbeing
	2. a sense of control over their: -health situation -isolation situation -day-to-day living in isolation via the mechanisms: shared decision making providing (1) personal items or (2) a single room	A [27] B [4,23,24,25,27,28,29]	
**Emotion-focused coping strategies**			
If older adults who experience contact isolation in a hospital setting during a pandemic and use emotion-focused coping strategies like:	trust in staff	[30,43]	This will impact their physical and psychological wellbeing
	2. positively evaluating conflict situations	[31]	
	3. seeking support from family/friends	[31]	
	4. increasing impulse control	[44]	

**Table 2:** Description of the setting.

Suspected COVID-19 Ward	Cohort COVID-19 Ward
**Admission:** Patients were awaiting the results of the COVID-19 test. After the diagnosis had been determined, the patient was transferred either to the cohort ward or regular wards.	**Admission:** Patients who tested positively for COVID-19
**Average stay:** 24 h	**Average stay:** 6 days
**Room situations:** (1) A single room with a dedicated bathroom. The room door had to remain closed, and the patient was not allowed to leave the room. Some single rooms had a staff entrance and changing room before the room. If the room was not equipped with a staff entrance the staff had to put on safety clothing in front of the room prior to entering.(2) A single room with a bathroom located in the hallway. The patient could leave the room to go to the bathroom but had to wear a face mask.	**Room situation:** Depending on availability, patients were placed in a single or a shared room. A staff entrance and changing room was at the entrance to the ward. The doors of the patient rooms could be left open. Patients could move more freely in the locked ward.
**Staff dress code:** Both on the suspected and cohort ward, all medical and cleaning staff wore protected disposable clothing on top of their normal work clothes including: a hair net, latex gloves, mouth-nose-masks (FFP2), goggles, and protective clothing.	

**Table 3:** Baseline characteristics of the study participants (*n* = 21).

Characteristics	Categories	*n* (%)
Sex	Male	14 (66.7)
	Female	7 (33.3)
Age (Years)	55–59	7 (33.3)
	60–69	6 (28.6)
	70 and older	8 (38.1)
Education	Low	4 (20)
	Middle	8 (40)
	High	8 (40)
Length of stay (days)	2–4	7 (33.3)
	5–7	9 (42.9)
	8–10	3 (14.3)
	>11	2 (9.5)

**Table 4:** Overview of the program theories about coping strategies in this specific context and setting.

Context	Mechanism	Explanation	Confirmed *	Outcome
	Problem-focused coping strategies			
**If** older adults experience contact isolation in a hospital setting during a pandemic **problem-focused** coping strategies are triggered by:	1. *approach of staff:* via the mechanisms: personalised approach Provision of information to the patient NEW: Professional competences of staff NEW: Provision of information from staff to relatives	A. Attention to patients on a personal level; taking time to sit on or next to the bed of the patients to talk with them about their feelings of reassure them -Providing own initiative for (care) tasks, reciprocal conversations with patients about topics not related to caregiving	✔	This will impact their physical and psychological wellbeing
		B. -Open and honest communication by the staff about the (health) circumstances of the patients	✔	
		C. -Professional approach of staff and regularly checking upon the patients	✔	
		D. -The awareness that relatives were kept up to date about the health situation of the patient by the staff	✔	
	2. are given a *sense of control* over their: -health situation -isolation situation -day-to-day living in isolation via the mechanisms: shared decision making NEW: Patients taking initiative for shared decision making Providing (1) personal items or (2) a single room NEW: increased recognisability of staff	-The patients feel in control of their health or day-to-day living situation -The patients understand the isolation situation -Conversely, some patients trusted and relied on the staff with decisions about their health, and did not express the need to be in control of their own health situation The patients feel in control of their health or day-to-day living situation -Staff listen to the needs of patient -Staff try to actively involve patients 1. Personal items: patients have more opportunities to control their day-to-day living situation because they are in charge of what they wanted to see or hear. 2. Single room: this theory could not be confirmed or refuted by our findings - Positive: create a calm environment for patients in which patients could focus on their own health - Negative: loneliness Staff could be less anonymous by wearing name tags or photos of themselves on their clothes	✔ ~ ✔ ✔ ~ ✔	
	**Emotion-focused coping strategies**			
**If** older adults experience contact isolation in a hospital setting during a pandemic and use **emotion-focused** coping strategies:	1. trust in staff	- professional care provision; - regular monitoring; - attention of staff; - availability of staff	✔	This will impact their physical and psychological wellbeing
	2. positively evaluate conflict situations	No explanation	−	
	3. seeking support via the mechanisms: (A) seeking support from family/friends (B) NEW: seeking support from fellow patients	Give patients strength and reassure them - Patients feel less isolated and lonely Realisation that the patient is not alone	✔ ✔	
	4. increased impulse control	No explanation	− −	
	5. NEW: Acceptance and rationalisation	- Patients understand the situation - Patients can get past the negative feeling	✔	
	6. NEW: Downward comparison	- Stimulated patients to think more positively about their own situation	✔	

* ✔ = Theory confirmed; ~ = Theory could not be confirmed nor be refuted; **− =** Theory not confirmed.

## Data Availability

Restrictions apply to the availability of these data. Data was obtained from and are available from Lenny M.W. Nahar–van Venrooij, l.nahar@jbz.nl; with the permission of the Jeroen Bosch Academy Research, Jeroen Bosch Hospital.

## Appendix A

**Table A1:** Semi-structured interview guide.

	Semi-Structured Interview Guide
Admission:	You were admitted to the hospital with symptoms. Could you tell me how your admission went and how you experienced it?
Closer look at health care providers (e.g., medical specialists nurses and paramedics)	How did you experience the contact between you and the care professionals (*Creating a therapeutic culture*)? How would you describe the skills of care professionals (including professional, information provision, commitment/motivation, conversation skills)? (Appropriate skill mix) (*Professional Competent*) and (*professional professionalism*) and (professional responsiveness) (*Interpersonal Skills*) and (*professional professionalism*) (*Commitment to the job*) To what extent were you involved (included/informed/ encouraged) to have a say in (e.g., treatment/medication) the care that was provided? (How did you perceive decisions made about your care within the care setting?) (Shared Decision making systems) (*Sharing decision-making*) and (*patient autonomy*) (*Having sympathetic presence*) (*professional compassion*) (*Clarity of Beliefs and values*) and (*Interaction Partnership*) (*Involvement with care*) (Interaction empowerment) (Effective staff relationships) (interaction partnership)
Care environment	If applicable, how did you experience the COVID suspect ward and/or cohort ward? To what extent was the environment (you were in) supportive? (Supportive organisational systems) How did you experience the isolation measures that applied to your situation? (Potential for innnovation and risk taking) How did you experience the hospital environment in which you were admitted?
Feelings during care moments	What was your well-being (mental and physical) during the care moments (How did you feel)? (*Feeling of well-being*) How did you experience the involvement of the care professionals? (*Engagement*) (*professional compassion*) To what extent were your personal needs and your opinion considered? (*Working with patients beliefs and values*) and (*patient uniqueness*) How was contact with your loved ones/how did you experience this? How did you experience the rules regarding the visitation arrangements? How did you experience the physical care provided by the care professionals (e.g., nutrition/care/nighttime/medication/oxygen)? (*Providing for physicial needs*)
Concluding questions on received care, consequences, discharge and tips	Overall, how did you experience the care (and the care provided by the care professionals)? What did you like, and what did you dislike? (*Satisfaction with care*) (*Knowing ‘Self’*) What effects/results did you experience from the care obtained? (outcomes) How was your discharge procedure? (e.g., the provision of information)? How did you experience this? What suggestions would you like to give the hospital?
